# Physicochemical and Antibacterial Evaluation of TiO_2_/CNT Mesoporous Nanomaterials Prepared by High-Pressure Hydrothermal Sol–Gel Method under an Ultrasonic Composite Environment

**DOI:** 10.3390/molecules28073190

**Published:** 2023-04-03

**Authors:** Huansheng Lai, Zilong Zhao, Wenhe Yu, Yuan Lin, Zhiyuan Feng

**Affiliations:** 1 Sino-French Institute of Nuclear Engineering and Technology, Sun Yat-Sen University, Zhuhai 519082, China; 2 School of Chemical Engineering and Technology, Sun Yat-Sen University, Zhuhai 519082, China; 3 Beijing Key Laboratory of Materials Utilization of Nonmetallic Minerals and Solid Wastes, National Laboratory of Mineral Materials, Engineering Research Center of Ministry of Education for Geological Carbon Storage and Low Carbon Utilization of Resources, School of Materials Science and Technology, China University of Geosciences, Beijing 100083, China; 4 Technical Center, Taiyuan Iron & Steel (Group) Co., Ltd., Taiyuan 030003, China; linyuan@tisco.com.cn

**Keywords:** carbon nanotubes, TiO_2_, mesoporous composite, bactericidal properties

## Abstract

TiO_2_ has attracted significant research interest, principally due to its nontoxicity, high stability, and abundance. Carbon-doped TiO_2_ can improve light absorption efficiency. In order to prepare high-efficiency photocatalysts, carbon-doped composites were prepared by hydrothermal reaction in a high-pressure reactor, and then TiO_2_/CNT mesoporous composites were prepared by the sol–gel method in an ultrasonic environment. Characterized by SEM and TEM, the composite materials contained TiO_2_ nanoparticles as well as CNT. After phase analysis, it was the anatase-doped phase. The following infrared light absorption performance and *Escherichia coli* bactericidal performance tests showed that it had better infrared and visible light absorption performance than pure TiO_2_. The TiO_2_/CNT mesoporous nanomaterials synthesized in this work are possible for clean industrial productions.

## 1. Introduction

With the advent of the steam engine revolution, electrical revolution, and information technology revolution, human beings have advanced by leaps and bounds in intelligent manufacturing, life sciences, and information technology [1,2,3,4,5]. However, with the development of science and technology, human beings also consume a lot of natural resources. In particular, the depletion of fossil non-renewable energy and environmental pollution poses new challenges to the development of science and technology [6,7,8,9,10,11].

Global climate change has prompted researchers from across the world to work together to search for new technologies for producing clean and renewable energy [12,13,14,15]. Photocatalytic reactions on semiconductive TiO_2_ are widely used and investigated for (1) water splitting in view of H_2_ production and (2) the destruction of environmental pollutants from water and air [16]. Starting from a point of practical engineering application, energy-efficient photocatalysts are highly demanded to effectively utilize the visible light that constitutes 43% of the total sunlight [17]. Hence, it is important to develop a visible light-responsive photo-catalyst, and some efforts have been devoted to developing “second-generation” TiO_2_ and other narrow band-gap semiconductors that can absorb visible light [18].

Numerous scientists have made gratifying achievements in the field of semiconductor materials. In particular, the large-scale and low-cost production of titanium dioxide P25 has the advantage of integrating various excellent properties in terms of material activity, specific surface area, and photoelectric performance [19,20,21,22,23]. The discovery of graphene is a gratifying achievement for mankind. The excellent properties of graphene have attracted research by scientists in various fields around the world and have made considerable achievements. However, the manufacturing cost of graphene has always been the bottleneck limiting its development. Therefore, many scientists have conducted research on carbon materials. From diamond and graphite, to fullerenes, carbon nanotubes, etc., carbon materials are full of magic. The development of 3D carbon-based nanocomposite multifunctional membranes has attracted researchers all over the world. The understanding of cavity quantum effect diffusions, reaction/inter-reaction, surface-area-to-volume ratio, and reaction kinetics is still at a preliminary level. Although we are able to produce a 3D nanotube filtration membrane at a thickness of around 200 nm, the further reduction in the thickness of this membrane for the fabrication of the membrane still remains a challenge. Therefore, it is important to search for new materials to fabricate the membrane. It has been reported in the literature that the combination of carbon materials and titanium dioxide can prepare excellent nanocomposite functional materials and exhibit various excellent properties. Especially in the fields of energy and environment, human beings urgently need to find sustainable energy materials and environmental governance materials [18,24,25,26]. However, the interfacial bonding performance of composite materials has always been a key factor affecting their development, and the composite interface of titanium dioxide and carbon materials in particular has always attracted the research of scientists.

In this paper, the preparation of titanium dioxide nanoparticles coupled with carbon nanotube composites by the high-pressure hydrothermal reaction was studied. Nano-mesoporous thin-film materials with a high specific surface area were prepared by the sol–gel method in the environment of ultrasonic radiation. The photoelectric properties and bactericidal properties of the composites were tested. The interface structure of the material was characterized by STEM in order to clarify the interfacial recombination mechanism of titanium dioxide and carbon nanotubes.

## 2. Experimental Procedure

### 2.1. Materials and Methods

First, commercial P25 TiO_2_ particles (InnoCHEM, average particle size = 25 nm) were blended with 5 wt.% multi-wall carbon nanotube (Shanghai, average particle size = 10–20 nm). The carbon-doped composites were prepared by hydrothermal reaction in an autoclave (150 °C, 6 h) using ethanol as solvent. After filtration, the obtained carbon-doped composite was dried in a vacuum drying oven (Jinghong, 80 °C, 2 h).

Tetra-n-butylorthotitanate [Ti(O-i-C4H9) 4] (TB) and ethanol absolute (EtOH) were used to prepare the Ti-bases sol. The addition of HCl was added to facilitate the hydrolysis rate of the mixture. The mole ratio of the ingredients was optimized at TB/EtOH = 1:50 with respect to the size of the nanoparticle produced by the sol.

The carbon-doped composite was dispersed into the Ti-based sol at 0.2 g/mL under vigorous magnetic stirring and subsequently placed in the ultrasonic bath for 5 h. The temperature was maintained at 80 °C. The sonicated mixture was then dried overnight at 80 °C and then calcined in an argon furnace (450 °C, 6 h) for converting the amorphous TiO_2_ into the anatase phase.

**Scheme 1:**
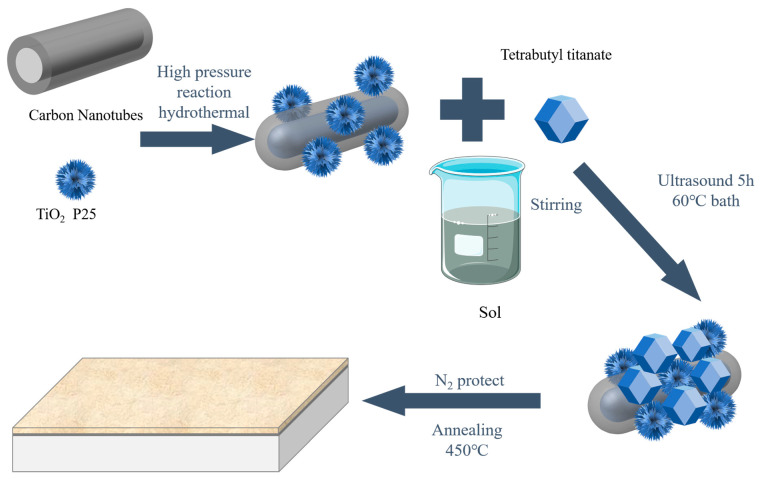
Schematic illustration of the formation of mesoporous TiO_2_/CNT nanomaterials membrane by sol–gel ultrasonic irradiation method.

The schematic diagram for material preparation is shown in Scheme 1.

### 2.2. Performance and Characterization

*Escherichia coli* was selected for bactericidal performance. An amount of 10 mg of the sample was added to 10 mL of deionized water (18.2 M Ohm) and sterilized in an automatic high-temperature sterilizer. After 1.15 g/L of the bacterial solution was diluted to 0.01%, 100 μL was added to TiO_2_ sterilization solution and irradiated under 34.4 klux sunlight for 1 h to prepare solid medium (trypsin 10 g, yeast powder 5 g, NaCl 10 g, agar 20 g and deionized water to prepare 1 L solution). Then, the sterilizing solution after illumination was applied to the medium, and the sterilization results were observed after 8 h.

A scanning electron microscope (SEM, JSM-7800F, JEOL, Tokyo, Japan) equipped with an electron backscattered diffraction system was employed for surface characterization. A transmission electron microscope (TEM, JEOL-2100F, Tokyo, Japan) was used for the structural analysis of TiO_2_. Phase analysis was carried out on X-ray diffraction (XRD, XPERT-PRO, Hawaii, HI, USA).

## 3. Results and Discussion

**Figure 1:**
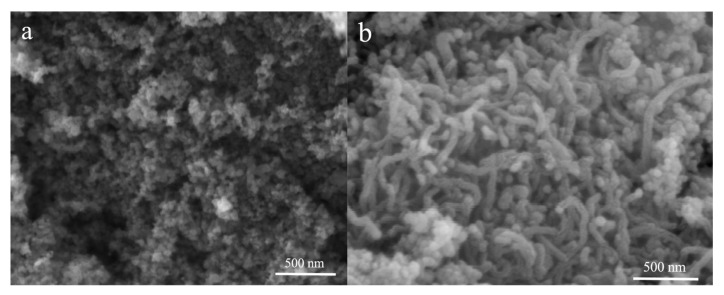
SEM micro-morphologies of the TiO_2_ (**a**) and TiO_2_/CNT (**b**) mesoporous nanomaterials.

**Figure 2:**
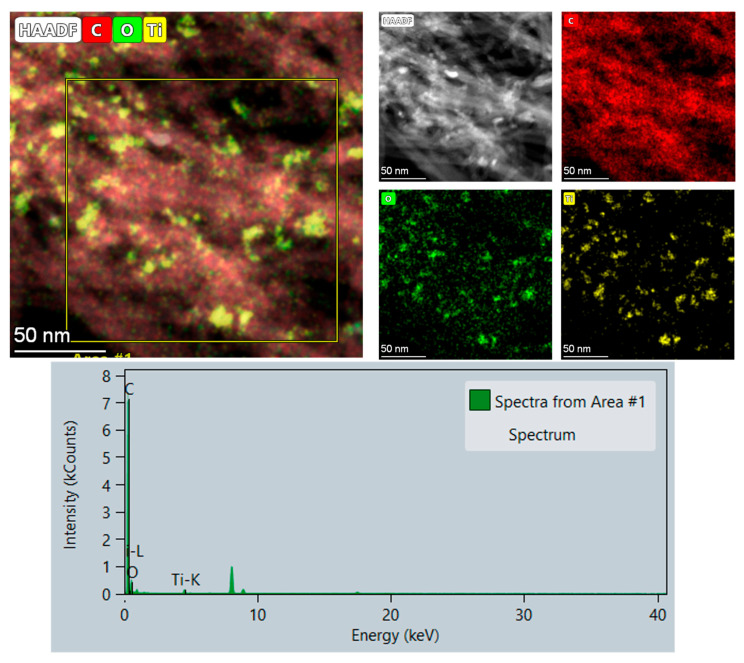
Energy-dispersive spectroscopy (EDS) mapping of the TiO_2_/CNT mesoporous nanomaterials.

SEM images of the TiO_2_ and TiO_2_ + CNT mesoporous composite are presented in Figure 1. The TiO_2_ prepared by the sol–gel method under ultrasonic irradiation was a mesoporous material (Figure 1a). Carbon nanotubes were rolled sheets of carbon atoms that form cylinders. After adding CNTs, TiO_2_ aggregated grow on the surface of carbon fibers and TiO_2_ nanoparticles were bonded around CNTs to form nanocomposites (Figure 1b). Moreover, the CNTs were exposed, confirming their successful incorporation into the CNTs. According to the energy dispersive spectroscopy (EDS) mapping in Figure 2. The results confirmed the presence and distribution of C, O, and Ti. The aggregated particles consisted of TiO_2_, and the strip structures consisted of CNTs. The green (O)- and yellow (Ti)-colored particles represented the TiO_2_ nanoparticles; the red (C)-colored structure represented CNTs. TiO_2_ nanoparticles were uniformly distributed on carbon nanotubes, which indicated the bonding between TiO_2_ nanoparticles and CNTs. It can be concluded that the one-dimensional CNTs were interlaced to form nanoporous structures, and zero-dimensional TiO_2_ particles were adhered to one-dimensional CNTs through gelation and sintering to form mesoporous composites. The agglomeration of the mesoporous materials was reduced by the ultrasonic process.

**Figure 3:**
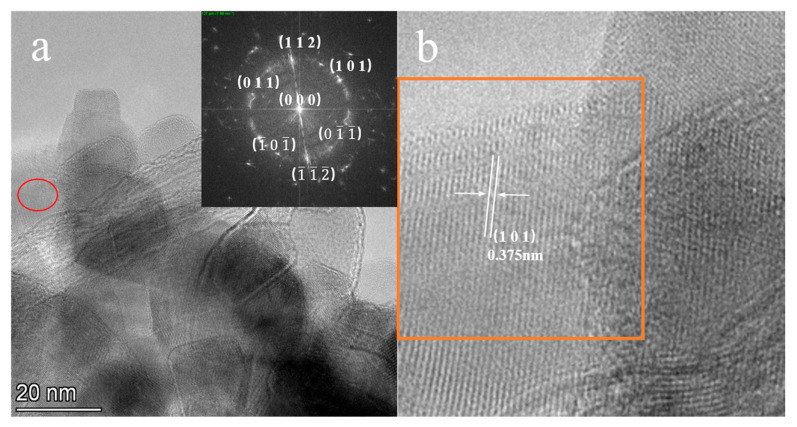
TEM (**a**) and high-resolution TEM (**b**) images of the TiO_2_/CNT mesoporous nanomaterials.

The microstructure of TiO_2_ on CNTs was examined by TEM, which revealed different crystal planes of the composite (Figure 3a). After magnifying the red-circle area in Figure 3a, more detailed information is presented in Figure 3b. The (101) interplanar spacing was 0.375 nm (Figure 3b), indicating that TiO_2_ was an anatase phase [27,28], and the TiO_2_ semiconductor composite on CNT improved the light absorption efficiency of the semiconductor composite [29].

**Figure 4:**
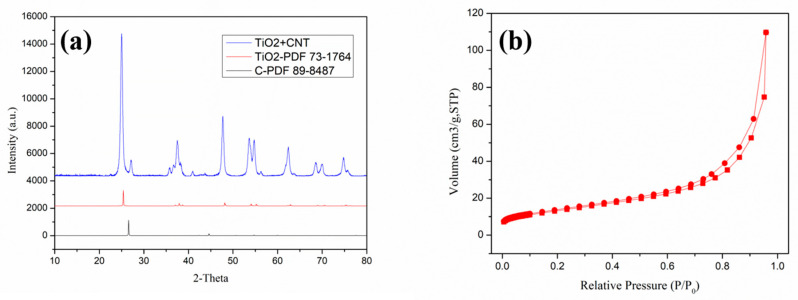
XRD pattern (**a**) and nitrogen adsorption–desorption isotherms (**b**) of the TiO_2_/CNT mesoporous composite.

According to the XRD results of the TiO_2_/CNTs mesoporous composite (Figure 4a), the XRD PDF cards of TiO_2_ and C were added for comparison. The TiO_2_/CNT mesoporous composite was an obvious anatase phase and C phase, which was conducive to improving the photoelectric properties of the material [30,31,32]. This result corresponds with the observation in SEM and TEM. As shown in Figure 4b, the BET specific surface area of TiO_2_/CNTs materials was 47.9 cm^3^/g, indicating the mesoporous structure. The high specific surface area was due to less agglomeration. When the relative pressure P/P_0_ is between 0.4 and 1.0, there is a hysteretic loop due to mesoporous adsorption, but the latter part of the curve bulged again, which corresponds to the porous adsorption system. Therefore, the dispersibility of TiO_2_ on CNTs is good.

**Figure 5:**
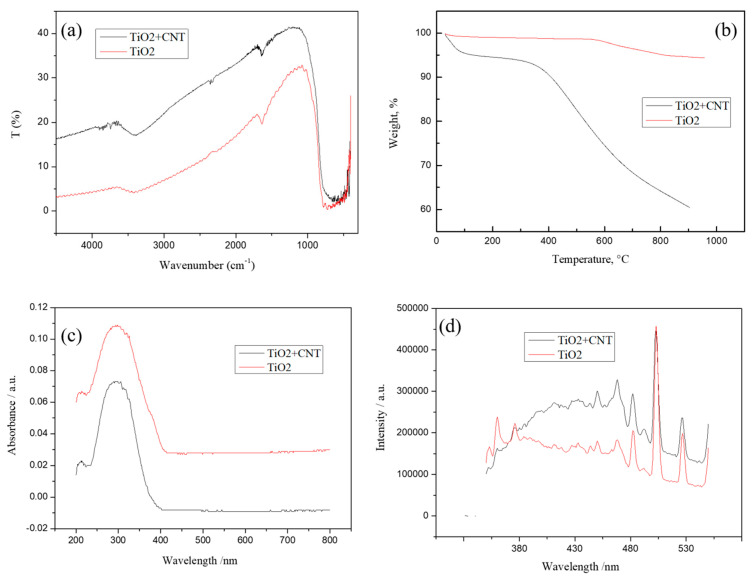
FT-IR spectra (**a**); thermogravimetric analysis (**b**); UV–vis (**c**) and photoluminescence spectra (**d**) of the TiO_2_ + CNT mesoporous nanomaterials.

FT-IR was employed to analyze the functional groups on the film. The FTIR spectra in Figure 5 showed characteristic bands at 3400 cm^−1^ and 1630 cm^−1^ correspond to the surface water and hydroxyl group [33,34]. TiO_2_ anatase has stable ultraviolet light absorption performance, but it has low light absorption efficiency in visible light and infrared light [35,36,37]. In order to improve the light absorption efficiency, carbon-doped TiO_2_ nanomaterials were prepared [38,39]. It can be seen from Figure 5 that the infrared absorption efficiency of TiO_2_/CNT mesoporous composite was higher than that of pure TiO_2_, especially in the 600–800 nm band. Therefore, the carbon-doped TiO_2_ nanomaterials behaved with a better light adsorption efficiency than the sample without carbon-doped TiO_2_ nanomaterials.

**Figure 6:**
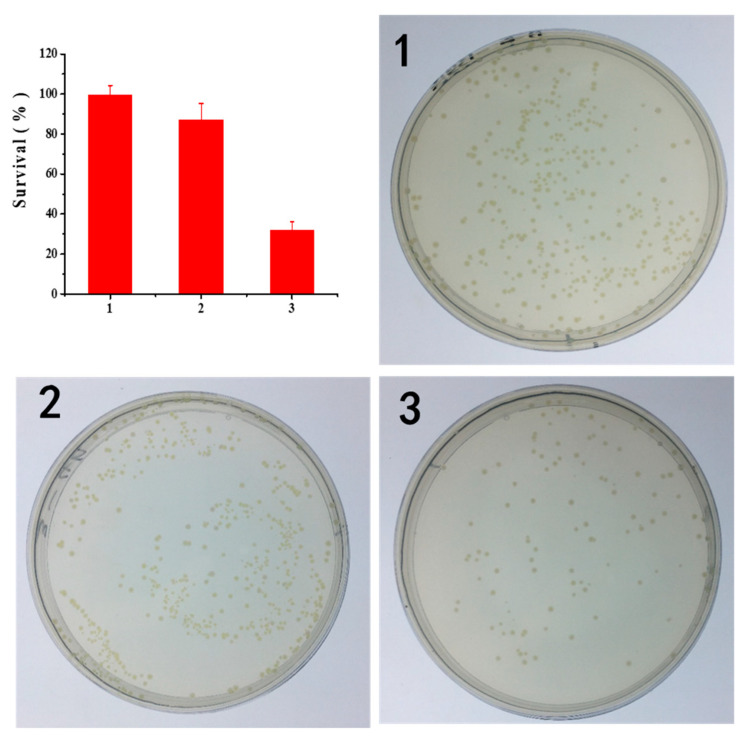
Bactericidal properties of the (**1**) black, (**2**) TiO_2_ mesoporous material, and (**3**) TiO_2_ + CNT mesoporous composite.

Biofouling including organic fouling on the conventional membrane surface is inevitable, which is caused by the nature of biological system, where microorganisms and bioparticles are the main components. The literature revealed that the structure/matrix of biofouling layer (biofilm) was highly related to the extracellular polymeric substances (EPS) compound. EPS is considered as the key component that determines the structural and functional integrity of microbial aggregates. It forms a three-dimensional, gel-like, highly hydrated and locally charged biofouling layer matrix, in which the microorganisms are more or less immobilized. In addition, EPS has also been reported to be the most significant foulant toward the conventional membrane fouling problems. Research has helped to address the problem, but it is still unclear what the major mechanism flaw is that causes biofouling, particularly on conventional membrane surfaces during the water treatment process. Currently, the industry practice to get rid of the biofilm is to take out the fouled membrane from the system and then soak it in chemicals such as sodium hypochlorite (NaOCl) and citric acid. This process causes three major problems: the generation of waste water, which requires further treatment; the interruption of the membrane treatment system, which reduces the production rate significantly; the reduction in membrane lifespan, which in turn increases the total cost of clean water production. The sterilization experiment embodies the photoelectric effect charge separation experiment. In order to test the visible light absorption and bactericidal performance of TiO_2_/CNT mesoporous composite, a mixture of *Escherichia coli* and different composites was made [40,41]. The mixture was spread on the agar medium after simulating sunlight irradiation, and the bactericidal results are shown in Figure 6. Figure 6(1) shows the blank group, and Figure 6(2) shows the bactericidal effect of TiO_2_ mesoporous material. TiO_2_ mesoporous material had a certain bactericidal effect, but TiO_2_/CNT mesoporous nanomaterials (Figure 6(3)) had a stronger bactericidal effect. The main reason was that the absorption efficiency of visible light was improved after doping CNT, and the stable composite interface is prepared under a high-pressure hydrothermal environment. Therefore, according to the results of bactericidal experiments, the TiO_2_/CNT mesoporous nanomaterials behaved with an excellent bactericidal performance, which is possible for clean industrial productions.

## 4. Conclusions

Preparation and stabilization of TiO_2_/CNT nanocomposites at the composite interface by a high-pressure hydrothermal method. TiO_2_/CNT mesoporous composite was prepared by the sol–gel method under an ultrasonic environment. Characterized under SEM and TEM, the composite materials contained TiO_2_ nanoparticles as well as CNT. After phase analysis, it was the anatase-doped phase. Infrared absorption performance and *Escherichia coli* bactericidal performance tests under visible light showed that TiO_2_/CNT mesoporous composite had better infrared and visible light absorption performance than pure TiO_2_.

## Data Availability

Not applicable.

## Sample Availability

Samples of the compounds are available from the authors.

## References

[1] Li H.L., Luo S.W., Zhang L.Q., Zhao Z.L., Wu M., Li W.H., Liu F.Q. (2022). Water- and Acid-Sensitive Cu_2_O@Cu-MOF Nano Sustained-Release Capsules with Superior Antifouling Behaviors. Acs. Appl. Mater. Interfaces.

[2] Pan J., Liu G., Lu G.M., Cheng H.M. (2011). On the True Photoreactivity Order of {001}, {010}, and {101} Facets of Anatase TiO_2_ Crystals. Angew. Chem. Int. Edit..

[3] Zhao Z.L., Li H.L., Huang L.X., Tan Y., Liu F.Q., Li W.H. (2021). Preparation of graphene quantum dots-doped TiO_2_ nanocomposites via a sol-gel method for photocathodic protection. Mol. Cryst. Liq. Cryst..

[4] Li J., Bai H., Feng Z. (2023). Advances in the Modification of Silane-Based Sol-Gel Coating to Improve the Corrosion Resistance of Magnesium Alloys. Molecules.

[5] Geng W., Wang L., Yang X.-Y. (2022). Nanocell hybrids for green chemistry. Trends Biotechnol..

[6] Zhao Z.L., Zhuang Y.L., Wang T., Zhao D.S., Wu X. (2022). Influence of Al-Si eutectic alloy on the mechanical behaviors and microstructure feature of ultralight dual-phase Mg-8Li-x(Al-12.6 Si) alloys. Mater. Today Commun..

[7] Zhao Z.L., Liu Y.D., Zhong Y.F., Chen X.H., Zhang Z.Q. (2018). Corrosion Resistance of as-rolled Mg-Li-AlSi Alloys. Int. J. Electrochem. Sci..

[8] Zhao Z.L., Li Y.H., Zhong Y.F., Liu Y.D. (2019). Corrosion Performance of as-rolled Mg-8Li-xAl Alloys. Int. J. Electrochem. Sci..

[9] Xue S.-G., Tang L., Tang T., Zhang F., Lyu H.-G., Liu H.-Y., Jiang J., Huang Y.-H. (2022). Identifying the active sites in C-N codoped TiO_2_ electrode for electrocatalytic water oxidation to produce H_2_O_2_. J. Cent. South Univ..

[10] Wang Y., Zhao X., Zheng Z., Jiang H., Chen T., Zhang Y., Cao H., Lin H., Zhan R. (2022). Pd-M-TiO_2_ (M=Mn, Cu, Ce and Fe) as passive NOx adsorber (PNA) at low temperature. J. Cent. South Univ..

[11] Zada A., Ali N., Subhan F., Anwar N., Ali Shah M.I., Ateeq M., Hussain Z., Zaman K., Khan M. (2019). Suitable energy platform significantly improves charge separation of g-C_3_N_4_ for CO_2_ reduction and pollutant oxidation under visible-light. Prog. Nat. Sci. Mater. Int..

[12] Hao D., Liu Y., Gao S., Arandiyan H., Bai X., Kong Q., Wei W., Shen P.K., Ni B.-J. (2021). Emerging artificial nitrogen cycle processes through novel electrochemical and photochemical synthesis. Mater. Today.

[13] Rade P.P., Giram P.S., Shitole A.A., Sharma N., Garnaik B. (2022). Physicochemical and in Vitro Antibacterial Evaluation of Metronidazole Loaded Eudragit S-100 Nanofibrous Mats for the Intestinal Drug Delivery. Adv. Fiber Mater..

[14] Wang S., Li J., Cao Y., Gu J., Wang Y., Chen S. (2022). Non-Leaching, Rapid Bactericidal and Biocompatible Polyester Fabrics Finished with Benzophenone Terminated N-halamine. Adv. Fiber Mater..

[15] Du Y., Hao Q., Chen D., Chen T., Hao S., Yang J., Ding H., Yao W., Song J. (2017). Facile fabrication of heterostructured bismuth titanate nanocomposites: The effects of composition and band gap structure on the photocatalytic activity performance. Catal. Today.

[16] Tripathy J., Lee K., Schmuki P. (2014). Tuning the Selectivity of Photocatalytic Synthetic Reactions Using Modified TiO_2_ Nanotubes. Angew. Chem. Int. Ed..

[17] Lee S.S., Bai H., Liu Z., Sun D.D. (2013). Novel-structured electrospun TiO_2_/CuO composite nanofibers for high efficient photocatalytic cogeneration of clean water and energy from dye wastewater. Water Res..

[18] Tang C., Bai H., Liu L., Zan X., Gao P., Sun D.D., Yan W. (2016). A green approach assembled multifunctional Ag/AgBr/TNF membrane for clean water production & disinfection of bacteria through utilizing visible light. Appl. Catal. B Environ..

[19] Zhao Z., Jiang X., Li S., Li L., Feng Z., Lai H. (2022). Microstructure Characterization and Battery Performance Comparison of MOF-235 and TiO_2_-P25 Materials. Crystals.

[20] Zheng H., Guo Y.-C., Shen F.-M. (2021). Production of titanium powder by metallothermic reduction of TiO_2_ in cold pressed pellets. J. Cent. South Univ..

[21] Zouheir M., Tanji K., Navio J.A., Hidalgo M.C., Jaramillo-Páez C.A., Kherbeche A. (2022). Effective photocatalytic conversion of formic acid using iron, copper and sulphate doped TiO_2_. J. Cent. South Univ..

[22] Yang F., Wen L., Peng Q., Zhao Y., Xu J., Hu M., Zhang S., Yang Z. (2021). Prediction of structural and electronic properties of Cl_2_ adsorbed on TiO_2_(100) surface with C or CO in fluidized chlorination process: A first-principles study. J. Cent. South Univ..

[23] Fan B., Liu H., Wang Z., Zhao Y., Yang S., Lyu S., Xing A., Zhang J., Li H., Liu X. (2021). Ferroelectric polarization-enhanced photocatalytic performance of heterostructured BaTiO_3_@TiO_2_ via interface engineering. J. Cent. South Univ..

[24] Xiang J., Wang S.C., Cao Y.X., Fang L.N., Ke W., Guo H., Duan B.Y., Yu W.H., Li L., Zhao Z.L. (2023). One-Step Preparation of High Performance TiO_2_/CNT/CQD Nanocomposites Bactericidal Coating with Ultrasonic Radiation. Coatings.

[25] Zhao Z., Lai H.S., Li H., Li L. (2021). Preparation and Properties of Graphene Doped TiO_2_ Mesoporous Materials for Photocathode Protection. Int. J. Electrochem. Sci..

[26] Zada A., Khan M., Hussain Z., Shah M.I.A., Ateeq M., Ullah M., Ali N., Shaheen S., Yasmeen H., Ali Shah S.N. (2022). Extended visible light driven photocatalytic hydrogen generation by electron induction from g-C_3_N_4_ nanosheets to ZnO through the proper heterojunction. Z. Phys. Chem..

[27] Wang Y.X., Rao L., Wang P.F., Shi Z.Y., Zhang L.X. (2020). Photocatalytic activity of N-TiO_2_/O-doped N vacancy g-C_3_N_4_ and the intermediates toxicity evaluation under tetracycline hydrochloride and Cr(VI) coexistence environment. Appl. Catal. B Environ..

[28] Wang C.J., Zhao Y.L., Xu H., Li Y.F., Wei Y.C., Liu J., Zhao Z. (2020). Efficient Z-scheme photocatalysts of ultrathin g-C_3_N_4_-wrapped Au/TiO_2_-nanocrystals for enhanced visible-light-driven conversion of CO_2_ with H_2_O. Appl. Catal. B Environ..

[29] Zhao J., Zhang J.L., Wang L., Lyu S.S., Ye W.L., Xu B.B., Qiu H., Chen L.X., Gu J.W. (2020). Fabrication and investigation on ternary heterogeneous MWCNT@TiO_2_-C fillers and their silicone rubber wave-absorbing composites. Compos. Part. A Appl. Sci. Manuf..

[30] Zhou W., Sun F., Pan K., Tian G., Jiang B., Ren Z., Tian C., Fu H. (2011). Well-Ordered Large-Pore Mesoporous Anatase TiO_2_ with Remarkably High Thermal Stability and Improved Crystallinity: Preparation, Characterization, and Photocatalytic Performance. Adv. Funct. Mater..

[31] Zhou W., Li W., Wang J.-Q., Qu Y., Yang Y., Xie Y., Zhang K., Wang L., Fu H., Zhao D. (2014). Ordered Mesoporous Black TiO_2_ as Highly Efficient Hydrogen Evolution Photocatalyst. J. Am. Chem. Soc..

[32] Xu H.M., Liu W., Cao L.X., Su G., Duan R.J. (2014). Preparation of porous TiO_2_/ZnO composite film and its photocathodic protection properties for 304 stainless steel. Appl. Surf. Sci..

[33] Hu G., Xiao Y., Ying J. (2021). Nano-SiO_2_ and Silane Coupling Agent Co-Decorated Graphene Oxides with Enhanced Anti-Corrosion Performance of Epoxy Composite Coatings. Int. J. Mol. Sci..

[34] Li J., Li T., Zeng Y., Chen C., Guo H., Lei B., Zhang P., Feng Z., Meng G. (2023). A novel sol-gel coating via catechol/lysine polymerization for long-lasting corrosion protection of Mg alloy AZ31. Colloids Surf. A Physicochem. Eng. Asp..

[35] Ghicov A., Albu S.P., Hahn R., Kim D., Stergiopoulos T., Kunze J., Schiller C.-A., Falaras P., Schmuki P. (2009). TiO_2_ Nanotubes in Dye-Sensitized Solar Cells: Critical Factors for the Conversion Efficiency. Chem. Asian J..

[36] Lu Y., Cheng X., Tian G., Zhao H., He L., Hu J., Wu S.-M., Dong Y., Chang G.-G., Lenaerts S. (2018). Hierarchical CdS/m-TiO_2_/G ternary photocatalyst for highly active visible light-induced hydrogen production from water splitting with high stability. Nano Energy.

[37] Nie X., Yin S., Duan W., Zhao Z., Li L., Zhang Z. (2021). Recent Progress in Anodic Oxidation of TiO_2_ Nanotubes and Enhanced Photocatalytic Performance: A Short Review. Nano.

[38] Asahi R., Morikawa T., Ohwaki T., Aoki K., Taga Y. (2001). Visible-light photocatalysis in nitrogen-doped titanium oxides. Science.

[39] Zheng J.Y., Lyu Y.H., Wang R.L., Xie C., Zhou H.J., Jiang S.P., Wang S.Y. (2018). Crystalline TiO_2_ protective layer with graded oxygen defects for efficient and stable silicon-based photocathode. Nat. Commun..

[40] Wu Q., Liu X.M., Li B., Tan L., Han Y., Li Z.Y., Liang Y.Q., Cui Z.D., Zhu S.L., Wu S.L. (2021). Eco-friendly and degradable red phosphorus nanoparticles for rapid microbial sterilization under visible light. J. Mater. Sci. Technol..

[41] Avsec K., Conradi M., Jenko M., Kocjancic B., Debeljak M., Gorensek M., Dolinar D. (2021). Effect of sterilization on the surface properties of ti6al7nb alloy femoral stems. Mater. Tehnol..

